# An AI-based nutrition recommendation system: technical validation with insights from Mediterranean cuisine

**DOI:** 10.3389/fnut.2025.1546107

**Published:** 2025-08-14

**Authors:** Kyriakos Kalpakoglou, Lorena Calderón-Pérez, Noemi Boqué, Metin Guldas, Çağla Erdoğan Demir, Lazaros P. Gymnopoulos, Kosmas Dimitropoulos

**Affiliations:** ^1^Visual Computing Lab (VCL), Information Technologies Institute (ITI), Centre for Research and Technology Hellas (CERTH), Thessaloniki, Greece; ^2^Technological Unit of Nutrition and Health, Eurecat, Technology Centre of Catalonia, Reus, Spain; ^3^Nutrition and Dietetics, Faculty of Health Sciences, Bursa Uludag University, Bursa, Türkiye; ^4^Biotechnology, Graduate School of Natural and Applied Sciences, Bursa Uludag University, Bursa, Türkiye

**Keywords:** artificial intelligence, AI-based recommender, personalized recommendations, nutritional recommendations, meal plan recommendations, healthy diet, Mediterranean cuisine

## Abstract

**Introduction:**

Modern lifestyle trends such as sedentary behaviors and unhealthy diets pose a major health challenge, as they have been related to multiple pathologies. Following a healthy diet has become increasingly difficult in today’s fast-paced world. Given this context, artificial intelligence can play a pivotal role in addressing the challenge.

**Methods:**

We present an AI-based nutrition recommendation system that generates balanced, personalized weekly meal plans tailored to the nutritional needs and preferences of healthy adults. The proposed method retrieves dishes and meals from an expert-validated database featuring Mediterranean foods, following a structured four-step process to recommend a weekly Nutrition Plan (NP).

**Results:**

The system’s performance is evaluated across 4,000 generated user profiles in three key areas: (a) dish/meal filtering accuracy based on user-specific parameters (e.g., allergies), (b) diversity of meals and food group balance, and (c) accuracy in caloric and macronutrient recommendations. The system achieves high accuracy in terms of suggested caloric and nutrient content while ensuring seasonality, diversity, and food group variety.

**Discussion:**

With solid accuracy in filtering, diversity, and caloric/macronutrient suggestions, the proposed system offers a promising solution to modern dietary challenges.

## 1 Introduction

Over the past few decades, sedentary behaviors and unhealthy dietary habits have emerged as significant public health challenges, contributing to the high prevalence of chronic diseases worldwide (1). The modern lifestyle, characterized by prolonged periods of inactivity, suboptimal physical activity, and the consumption of ultra-processed and/or high-calorie foods, poses a substantial risk to overall health and well-being (2). This trend towards sedentary behavior and unhealthy dietary intake has been associated with an increased incidence of obesity, cardiovascular diseases, type 2 diabetes, and other metabolic disorders (3, 4).

Despite growing awareness of the importance of balanced diet choices, many individuals struggle to adopt and maintain balanced dietary habits and regular physical activity routines (5). The widespread consumption of processed foods, coupled with work, family, and social demands, and the decline of traditional dietary patterns, often lead to the prioritization of convenience over health, resulting in suboptimal dietary choices and reduced physical activity (5, 6).

To tackle these challenges and encourage healthier eating habits, there has been a growing interest in developing nutrition recommendation systems, applications, and tools designed to offer personalized dietary guidance and recommendations to users. These applications leverage advances in technology, such as Artificial Intelligence (AI) and Machine Learning (ML), to analyze user data and preferences (e.g., dietary choices, cultural considerations) and deliver tailored recommendations for optimal nutrition and wellness (7). Latest approaches exploit the advantages of Large Language Models, such as ChatGPT (8), or use hybrid models based on GenAI and ChatGPT to provide personalize meal plans (9).

More specifically, food and nutrition recommendation methods and systems can be broadly categorized into traditional food recommendation systems and AI-based nutrition recommendation systems (10). Traditional food recommendation systems utilize various techniques, including combinatorial optimization techniques (e.g., knapsack algorithm, integer and linear programming), content-based filtering, collaborative filtering, and hybrid approaches. Combinatorial analysis in meal planning optimizes food selection and meal sequencing by balancing nutritional requirements, cost efficiency, and user preferences (11, 12). In contrast, content-based, collaborative and hybrid techniques focus on correlating food item attributes and/or user preferences to provide personalized suggestions (13). AI-based nutrition recommendation systems focus on recommending meals, meal plans or diets, based on their nutritional content, as well as on the dietary restrictions and health goals of the user; these are in turn separated into knowledge-based and ML-based systems. Each of these approaches plays a crucial role in catering to the diverse needs and preferences of users in the realm of food recommendation.

Combinatorial optimization techniques, such as the knapsack algorithm, integer programming and constraint satisfaction problems, are used to generate meal plans by selecting optimal combinations of food items while ensuring dietary diversity, adherence to food group intake rules, and compliance with user preferences and restrictions (14, 15). Another combinatorial technique, such as linear programming (LP) in meal planning, starting from (16), formulates meal planning as an optimization problem where the objective is to either minimize cost or maximize nutrient intake while adhering to essential dietary guidelines, caloric requirements, and promoting food diversity (17, 18). Although combinatorial techniques offer mathematically optimal solutions for nutrition planning, they often fall short in accommodating user goals and preferences, complex nutritional rules, and food diversity—factors essential for real-world meal planning (18, 19).

Other traditional recommendation techniques, include content-based, collaborative-based, and hybrid methods (20, 21). Content-based filtering focuses on finding food items that match the preferences of a user profile or are similar to items the user has interacted with previously (22, 23). Collaborative filtering, on the other hand, relies on similarities between user profiles to generate food recommendations. By analyzing user behavior and preferences, collaborative filtering identifies users with similar preferences and recommends items that these similar users have enjoyed (24, 25). Hybrid methods combine aspects of both content-based and collaborative filtering to produce more accurate and diverse recommendations (26). By leveraging the strengths of each approach, hybrid methods aim to overcome the limitations of individual techniques and provide users with more personalized and relevant food recommendations.

Partly due to the limitations of traditional food recommendation systems, e.g., data sparsity, the cold start-problem, scalability (27), and mainly due to a growing need for recommender systems that focus on user needs and complex nutritional guidelines, new recommendation techniques have emerged. AI-based nutrition recommendation systems represent a shift towards generating recommendations that prioritize health and wellness considerations, offering personalized suggestions based on users’ preferences and nutritional requirements. These systems can be grouped into knowledge-based and ML-based systems. In the realm of knowledge-based systems, the traditional recommendation approach can switch, for example, to a many-objective optimization (MaOO) approach, providing a more balanced way of recommending meals incorporating attributes such as user preferences, nutritional values, dietary diversity, and user diet patterns (28). Another example is the Meal Plan Generator (MPG) mechanism (29), that can synthesize meals from foods. Considering many factors such as caloric intake, food preferences, variety, and compatibility, the MPG mechanism can provide users with meal proposals generated from a given set of foods. As recommendations become more complex, systems like the Protein AI-advisor (30) can generate weekly meal plans according to each user’s profile and preferences, expert-validated rules, and food diversity criteria. Meals, already curated by nutritionists, are synthesized to create daily nutritional plans that closely align with users’ needs combined with rules. On the other hand, machine learning-based systems leverage advanced algorithms and data analysis techniques to provide personalized recommendations by learning from user behavior, preferences, and contextual information. In (31), the AI-based diet recommendation engine leverages a deep generative network to deliver daily personalize meal plans tailored to users’ needs. Additionally, limitations in the meal databases are addressed by incorporating ChatGPT to generate equivalent alternative meals.

Most of the existing approaches focus on specific factors such as the user profile and preferences, or caloric and macronutrient consumption. However, to produce even more personalized meal plans, an AI recommender should consider simultaneously various criteria. To this end, in this paper we present a novel approach which combines multiple factors, such as user profile and preferences, local cuisine choices, seasonality, expert nutritional rules, daily and weekly food group criteria and diversity, to propose weekly meal plans based on a Mediterranean database, while still achieving high accuracy in terms of energy intake and macronutrients.

In particular, the proposed mechanism takes into consideration user information such as allergies, preferences, and local cuisine to retrieve appropriate meals from an expert-validated database, featuring Mediterranean foods. Seasonality is used as an extra filter to select the right meals for the user. Subsequently, synthesis of all possible daily Nutrition Plans (NPs) is performed, and the algorithm sorts them according to the Daily Energy Requirement (DER) of the user and according to appropriate rules provided by expert nutritionists (knowledge base). DER is calculated from the physical characteristics of the user (sex, age, weight, height, Physical Activity Level or PAL). After ensuring food group variety and diversity, seven daily Nutritional Plans (NPs) are proposed to the user (weekly Dietary Plan or weekly Nutritional Plan).

The main contributions of this work are summarized as follows:

- A novel AI-based nutrition recommender (AINR) for balanced personalized meal plans is proposed. The AI recommender considers multiple factors to improve personalization.
- A new expert-validated Mediterranean meals and dishes database is presented. The database was designed and implemented with meal options from two Mediterranean countries (Spain and Türkiye).
- Finally, a thorough evaluation of the AI-based nutrition recommender (AINR) was conducted using 4,000 generated user profiles.

The work was conducted within the PRIMA SWITCHtoHEALTHY project (32).

## 2 Materials and methods

### 2.1 AI-based nutrition recommender (AINR)

The proposed system aspires to empower individuals towards healthier dietary choices. In this context, the proposed AINR was designed to provide personalized weekly Nutritional Plans (NPs) to users, based on their profile and preferences, the principles of a healthy diet, and a plethora of Mediterranean dishes/meals.

*User profile and preferences*. The user profile contains four types of nutrition-related user data: personal information, physical characteristics, physical activity level, and allergies, as well as three types of user preferences: cultural preferences, cuisine preference, and user interface language preferences. Table 1 provides more details on the user profile/preferences data.

**Table 1:** User profile/preferences data.

	Data input → ↓ Data type	Provided by the user	Calculated by the system
User profile	Personal information	Username Password Email	–
	Physical characteristics	Sex [*Male*, *Female*] Year of birth (YB) Height (H), in m Weight (W), in kg	Body Mass Index (BMI) Basic Metabolic Rate (BMR)
	Physical activity level	[*Sedentary (=1.2)*, *Lightly active (=1.375)*, *Moderately active (=1.55)*, *Very active (=1.725)*, *Extra active (=1.9)*]	Daily Energy Requirements (DER)
	Allergies	[*Milk protein*, *Eggs*, *Fish/Seafood*, *Nuts*]	–
User preference**s**	Preferences	[*Halal*]	–
	Cuisine	[*Spanish*, *Turkish*]	–
	Interface language	[*English*, *Spanish*, *Turkish*]	–

*Healthy diet principles.* Concrete rules regarding the calculation of nutrition-related attributes (i.e., BMI, BMR, DER), as well as the recommended dietary consumption in terms of energy and macronutrients balance, food groups, and food variety, have been provided by nutrition experts. These rules are presented and discussed in detail in Sections 2.1.3 and 2.1.4.

*Mediterranean dishes and meals*. Nutrition experts have provided a significant number of Mediterranean meals and corresponding dishes. Specifically, detailed information regarding the food composition and attributes of 180 meals, 90 from Spanish cuisine and 90 from Turkish cuisine, has been provided. The database of Mediterranean dishes and meals is presented and analyzed in detail in Section 2.2.

The personalized Nutritional Plans (NPs) are generated through a structured multi-step process, as outlined below.

Step 1 *Consider meal seasonality/local cuisine*. Meals are filtered out based on the seasonality and the locality of the ingredients they contain (see Section 2.1.1 for details).

Step 2 *Filter inappropriate meals*. Meals that are not appropriate for a specific user profile are excluded, e.g., meals containing allergy agents or foods conflicting with the preferences of the user (see Section 2.1.2 for details).

Step 3a *Create daily NPs*. All possible combinations of the remaining meals are calculated and stored as daily NPs consisting of five meals: *breakfast*, *morning snack*, *lunch*, *afternoon snack*, *dinner* (see Section 2.1.3 for details).

Step 3b *Short daily NPs*. All daily NPs generated in the previous step are sorted from optimal to suboptimal based on the dietary requirements of the user as these are calculated via expert validated rules (see Section 2.1.3 for details).

Step 4a *Consider food group intake balance*. The ordered Nutritional Plan (NP) list generated in the previous step is used to create a weekly NP (consisting of seven daily NPs), ensuring appropriate consumption of various food groups, aligned with the principals of a healthy diet. (See Section 2.1.4 for details).

Step 4b *Ensure dish/meal diversity*. In parallel to the previous step, the recommender ensures sufficient diversity of dishes and meals in the weekly Nutritional Plan (NP) for a pleasurable and sustainable diet. (See Section 2.1.4 for details).

**Figure 1:**
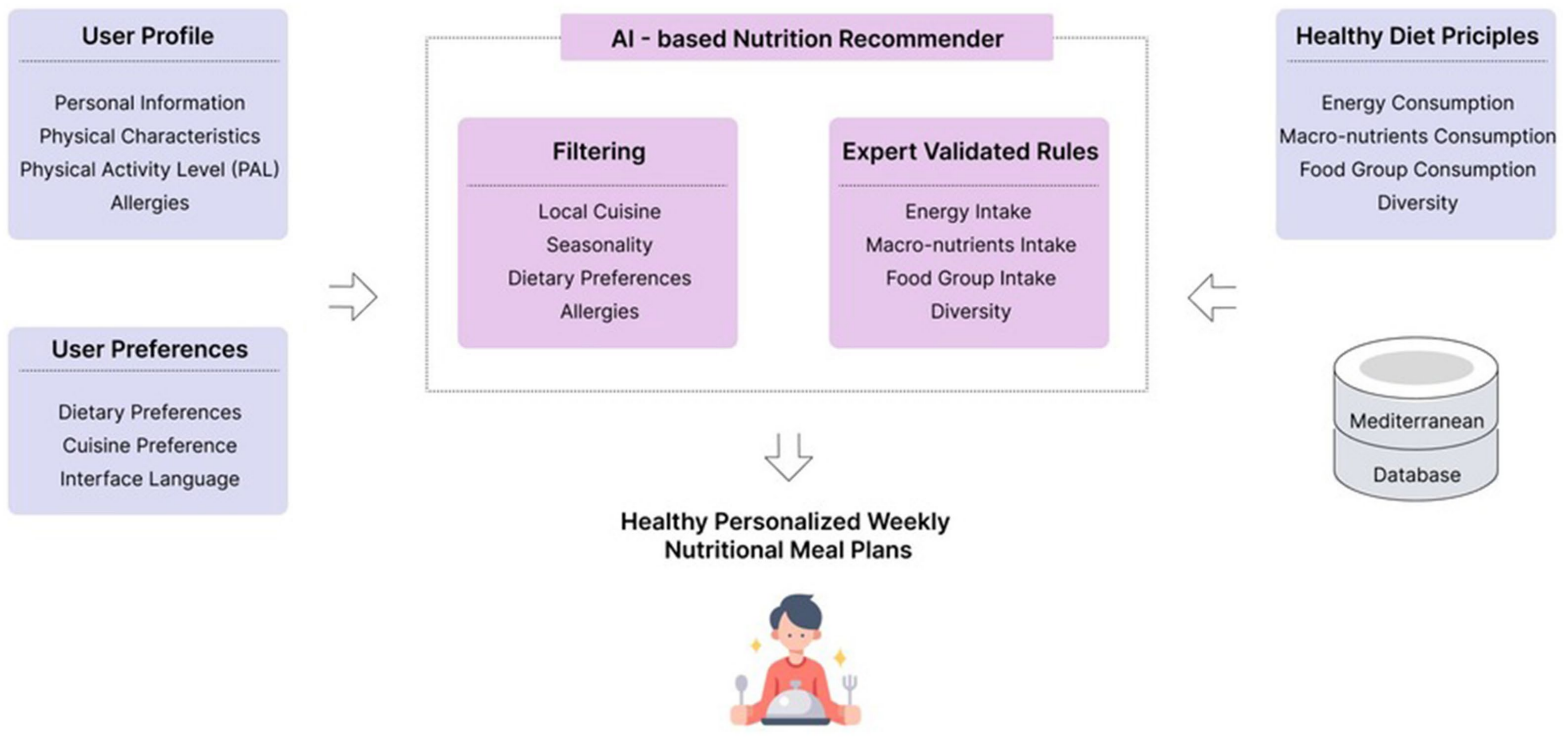
AI-based nutrition recommender (AINR). The AINR is fed with the user’s profile and preferences, with healthy diet principles, and foods from a Mediterranean meals and dishes database. The core of the AINR processes this information using expert-validated rules and filtering mechanisms to propose healthy personalized weekly meal plans to users.

In Figure 1, the AI-based nutrition recommender (AINR) mechanism illustrated.

#### 2.1.1 Considering meal seasonality/local cuisine

Consuming seasonal and local foods offers substantial health benefits, as produce harvested in-season tends to retain higher levels of vitamins, minerals, and antioxidants. It also reduces environmental impact by lowering the carbon footprint associated with long-distance transport and energy-intensive storage, and supports local economies and biodiversity by promoting regional agriculture. Additionally, seasonal eating helps reduce food waste by aligning consumption with natural production cycles and can lead to significant cost savings. These principles are in line with sustainable healthy diet guidelines (33). Meals with seasonal and local ingredients are often more appealing due to their superior freshness, flavor, and nutritional value. Therefore, we designed the system database to include seasonality and locality information for dishes and meals (see technical details in Section 2.2), enabling a query mechanism that retrieves meals tailored to each season (winter, autumn, summer, spring) and local cuisine (Spanish, Turkish).

#### 2.1.2 Filtering inappropriate meals

When it comes to food suggestions, it is crucial to ensure that the proposed meal plans do not contain ingredients that are conflicting with allergy indications in the user profile or with the user’s preferences. This is essential for safeguarding user health and ensuring cultural sensitivity. For instance, individuals with nut allergies risk severe allergic reactions (anaphylaxis) if exposed to nuts. Similarly, diets such as halal or vegetarian restrict specific food groups for ethical, religious, or health-related reasons. Therefore, the exclusion of such meals is not merely preferential but critical for adherence to dietary constraints and user safety. Technically, this has been achieved by structuring the system database in a way that allows inclusion of information regarding allergens (dairy, eggs, fish, and nuts) or unwanted ingredients (pork in the case of halal diet) contained in meals. Each time a user updates their profile, a query mechanism filters out all inappropriate meal options, allowing only suitable ones to proceed to the next phase. Finally, users can specify whether they prefer meal suggestions from Spanish or Turkish cuisine.

#### 2.1.3 Creation and shorting of daily NPs

As presented previously, the proposed AINR has been meticulously designed to exclude meals based on user allergies and preferences, while also incorporating meals according to seasonality and locality. The remaining meals, after this initial filtering, are used to synthesize all possible daily NPs consisting of five meals of the type: breakfast, morning snack, lunch, afternoon snack, and dinner. Subsequently, the daily NPs are scored for the specific user based on their profile and appropriate expert validated rules.

It must be noted that the synthesized daily NPs are not explicitly evaluated by expert nutritionists since their number is in the hundreds of thousands. However, expert validated nutritional rules will be subsequently applied to these daily NPs to produce a suggested weekly NP (recommendation) and human oversight is strongly advised in any usage of the system with real users.

To effectively explain how our scoring system operates, we first introduce the key parameters required by the recommender and describe how they are derived from the user profile. The following section presents the formulas used in these calculations. Each parameter is defined alongside the formula used to compute it, and is subsequently applied in later calculations.

Age (**A**):


A=Current Year−YB,

where *YB* stands for the Year of Birth of the user.

Body Mass Index (**BMI**):


$$\mathrm{BMI}=W÷H^{2},$$

where *W* and *H* stand for the user weight and height, respectively. BMI is subsequently used for the calculation of the daily energy requirement (see below).

Basal Metabolic Rate (**BMR**) according to the Harris-Benedict equation (34):


BMR=88,362+(13,397×W)+(4,799×H)−(5,677×A),formen

$$\begin{array}{l} \mathrm{BMR}=447,593+(9,247\times W)+(3,098\times H)-\\ (4,330\times A),\text{for women} \end{array},$$

where *W*, *H and A* stand for the user weight, height and age, respectively. Note that our algorithm uses the Harris-Benedict equation to calculate the user BMR. Despite this equation has been validated and widely used in both clinical and healthy population (35) studies, we do not rule out exploring more recent methods such as the Mifflin-St Jeor equation in future validations to further improve the accuracy of our calculations.

Daily Energy Requirements (**DER**), (36, 37):


DER=(BMR×PAL)+500,ifBMI<18,5

DER=BMR×PAL,if18,5≤BMI≤24,99

DER=(BMR×PAL)−500,ifBMI>24,99,

where *PAL* stands for the Physical Activity Level of the user corresponding to the following values: Sedentary (=1.2), Lightly active (=1.375), Moderately active (=1.55), Very active (=1.725), Extra active (=1.9); BMI stands for the Body Mass Index; BMR stands for Basal Metabolic Rate. If the user’s BMI exceeds 30 (Obese), the daily energy requirements is reduced to promote weight loss, while for a BMI below 18.5, the daily energy requirements is increased to support weight gain. Within a healthy BMI range, daily energy requirements matches the user’s baseline energy needs.

For optimization purposes 100.000 daily NPs are randomly selected from the much larger pool of total generated daily NPs. Each plan then is scored based on two key factors: (i) the user’s DER, and (ii) expert-validated rules related to daily fat and protein (macronutrient), and fruit and vegetable consumption (details are provided in Table 2). Scoring serves as the basis for sorting the daily NPs from the more suitable (lowest score) to the less suitable (highest score) for a given user profile. The exact algorithm used to evaluate the suitability of daily NPs for a given user profile (i.e., scoring and sorting) is detailed below.

**Table 2:** Macronutrients and Fruit and Vegetable consumption boundaries for adults that are used for the sorting of the daily NPs.

Adults	Protein %DER	Fat %DER	Fruits & Vegetables
Male	15–20	25–40	5–10
Female	15–20	25–40	5–10

A Daily NP Score (**DNPS**) is calculated for each daily NP, using the following equation:


DNPS=CS+PS+FS+FVS,

where *CS*, *PS*, *FS*, and *FVS*, stand for the *Caloric Score*, *Protein Score*, *Fat Score*, and *Fruits and Vegetables Score* of the daily NP, respectively. Since protein and fat in *Caloric* and *Protein Scores* are measured as percentages of the user daily energy requirements, carbohydrates can be excluded from the equation, as they account for approximately the remaining percentage.

These four partial scores are calculated by the following equations.

Caloric Score (CS):


CS=∣DER−TK∣,

where *DER* is *Daily Energy Requirements* of the user and *TK* is the sum of Kcal in a daily NP.

This rule ensures that the proposed meals closely match the user’s calculated daily energy needs. Consistently under-consuming calories could lead to nutrient deficiencies and weight loss, while exceeding caloric needs contributes to weight gain and metabolic disorders. The closer the total caloric content of a daily plan is to the user’s DER, the more aligned it is with maintaining or adjusting body weight in a healthy way (38).

Protein and Fat Scores (PS, FS):


PS=0,if0,15≤TPP≤0,2

PS=50,ifTPP<0,15or0,2<TPP,

where *TPP* is the sum of protein in a daily NP as a percentage of the *DER*.


FS=0,if0,25≤TFP≤0,4

FS=50,ifTFP<0,25or0,4<TFP,

where *TFP* is the sum of fat in a daily NP as a percentage of the *DER*.

The dietary thresholds used in these equations have been selected according to the usual protein and fat intake in the context of the Mediterranean Diet; That is 15–20% of the daily energy intake coming from protein and 25–40% coming from fat, as reported in the bibliography (39, 40).

Fruits and Vegetables Score (FVS):


FVS=0,if5≤TFV≤10

FVS=100,ifTFV<5or10<TFV,

where *TFV* is the sum of all dishes in a daily NP that contain either fruit or vegetable.

A minimum of 5 servings of fruits and vegetables per day is a cornerstone of global dietary guidelines due to their protective role against chronic diseases like heart disease, diabetes, and certain cancers (38). More than 10 dishes containing fruit or vegetables could indicate impractical or unsustainable eating patterns. Hence, the system aims for a realistic, health-promoting range.

#### 2.1.4 Creation of weekly NPs

We established clear rules to ensure that the proposed meal plans comply with fundamental healthy diet principles related to food group intake and variety (41).

The AINR examines the sorted list of daily NPs, starting from the most appropriate, until it finds a proper combination of seven daily NPs that:

- Do not contain the ingredient “egg” more than once daily
- Do not contain any of the following ingredients daily for both lunch and dinner: “white meat,” “red meat,” “pork,” “fish or seafood,” “pulses,” “pasta,” “rice.”
- Do not contain any of the following ingredients more than three times per week: “tubers,” “rice,” “pasta,” “fish.”

These constraints are grounded in key dietary principles that emphasize variety, nutrient balance, and long-term health protection. Limiting certain foods such as tubers, rice, pasta, and fish to no more than three times per week prevents nutritional imbalances that could arise from over-relying on a limited set of ingredients. For instance, while fish is a valuable source of omega-3 fatty acids, frequent consumption may increase exposure to environmental contaminants like mercury (42). Similarly, staple foods like rice or pasta, when consumed excessively, may displace other nutrient-dense food groups (e.g., legumes, vegetables), reducing overall diet quality.

The restriction of egg consumption to no more than once daily follows a similar rationale. While eggs are nutrient-rich, providing high-quality protein and essential micronutrients, excessive intake – particularly when combined with other dietary sources of cholesterol – has been associated with increased cardiovascular risk in certain populations (43). These rules align with dietary patterns and international nutrition guidelines that advocate for moderation and diversity to support both metabolic health and sustainable eating habits.

As a final step, the AINR ensures dish and meal diversity by excluding daily NPs that:

- Contain the same dish more than once per day
- Lead to the repetition of the same dish more than three times per week
- Lead to the repetition of the same meal more than two times per week
- Lead to the repetition of the same meal sequence more than two times per week.

Repetition can reduce user engagement and satisfaction with the nutritional plan while at the same time reducing the variety of the diet. From a behavioral nutrition perspective, monotonous diets often lead to decreased adherence. Promoting diversity supports long-term commitment to healthy eating by maintaining meal appeal and preventing taste fatigue.

### 2.2 The SWITCHtoHEALTHY Mediterranean meals and dishes database

The SWITCHtoHEALTHY Mediterranean Meals and Dishes Database is a comprehensive repository of meals and dishes from two cuisines: Spanish and Turkish. Expert nutritionists from each participating country meticulously curated the database, adhering to the principles of the Mediterranean diet. Each country-specific database offers a wide variety of dishes and meals representative of the Mediterranean diet that are commonly consumed within each region. While the AINR primarily focuses on the user DER, proteins and lipids, the dishes and meals were designed to include a variety of nutrient-dense foods, such as fruits, vegetables, legumes, whole grains, and healthy fats, all of which are staples of the Mediterranean diet. Olive oil serves as the primary cooking fat, complemented by the use of herbs and spices to enhance flavors. The recipes also incorporate healthy cooking techniques while minimizing added sugars and processed foods. Additionally, local ingredients were prioritized to promote sustainability and authenticity in the menus. Finally, all recipes have been translated into English and the respective local language to ensure accessibility.

To develop the Spanish database, the dietary guidelines from the Public Health Agency of Catalonia were considered (44). To develop the Turkish database, the guidelines of the Turkish Ministry of Health and the Turkish Ministry of Agriculture and Forestry were reviewed, as well as the 2022 Türkiye Nutrition Guide published by the General Directorate of Public Health of Türkiye (45). A summary of the Mediterranean Diet features that were considered during the preparation of the meals and dishes is provided in Table 3.

**Table 3:** Summary of the Mediterranean Diet features for dishes and meals preparation.

Mediterranean diet feature	Application in the meal plans
Emphasis on plant-based foods	Conform the base of main meals, including raw or cooked vegetables, and fresh fruits. ***Frequency of consumption:*** *Vegetables:* Minimum 2 per day *Fresh fruits:* Minimum 3 per day
Healthy fats	Olive oil as the primary fat, nuts and seeds. ***Frequency of consumption:*** *Olive oil:* For dressing and cooking. 4–5 tablespoons Nuts and seeds: 3–7 handfuls per week
Whole grains	Whole grains like barley, oats, and wheat, often in the form of bread, pasta, and rice. Bulgur wheat mainly in Turkey. ***Frequency of consumption:*** At every meal
Legumes	High intake of legumes like beans, lentils, and chickpeas. In traditional dishes or in mixed salads with vegetables. ***Frequency of consumption:*** 3–4 times a week
Herbs and spices	High use of herbs and spices like garlic, basil, oregano, rosemary, thyme in the dishes.
Moderate fish and seafood	Moderate to high intake, especially oily fish like salmon, sardines, and anchovies. Provided as a semi-unique dish, usually combined with vegetables. ***Frequency of consumption:*** 3–4 times a week
Moderate dairy products	Primarily milk, cheese and yogurt (especially sheep and goat milk-based). ***Frequency of consumption:*** 1–3 times per day
Water as the main drink	It is recommended as the main source of hydration throughout the day, depending on thirst.
Emphasis on whole, natural foods	Use of minimal processed foods. Emphasizing whole, unprocessed foods, avoiding overly processed and refined products. Traditional recipes dominate.
Limited red meat and processed meat	Limited to occasional consumption, typically in small portions. Mainly provided in the form of cured meats (ham in Spain).
Seasonality	Meals prioritize seasonal produce, ensuring that fruits and vegetables are at their peak in terms of flavor, texture, and nutritional value. This also helps with food variety and sustainability.
Variety of colors	Wide variety of fruits and vegetables in the dishes, to ensure colorful meals with a rich array of antioxidants, vitamins, and minerals. A colorful plate often includes red, green, purple, yellow and orange colors.
Local ingredients	Using locally sourced ingredients reduces the environmental impact and supports local economies while ensuring the food is as fresh and nutrient-rich as possible. Encouraging the use of olive oils, cheeses, fresh fruits and vegetables, and seafood, all of which are key components of the diet.
Healthy cooking techniques	Emphasis on grilling, baking, steaming, and sautéing with olive oil instead of frying.

#### 2.2.1 Dishes/meals design

##### 2.2.1.1 Dishes

It contained a variety of dishes where the ingredients for their preparation were detailed including standard quantities for adults. In addition, the preparation method, the nutritional composition of a standard portion (Kcal, protein, fat, carbohydrates) and different tips related to the recipe were included. The dishes were identified with a numerical ID and were classified according to different factors such as seasonality (based on the ingredients and culinary preparation), type of food, food group and the colors of the main ingredients. As rabbit meat, pork, and plant-based dairy products are not widely consumed in Türkiye, they were minimally included in the Turkish database.

##### 2.2.1.2 Meals

The database includes combinations of dishes suitable for the main daily meals: breakfast, morning snack, lunch, afternoon snack, and dinner. Each meal (a combination of dishes) was carefully designed to ensure an optimal balance of nutrients and food groups. Table 4 provides an example of a Spanish daily meal plan, including four meals—Breakfast, Lunch, Dinner, and a Snack (instead of two snacks for simplicity)—along with their corresponding dishes, nutritional information, food composition and recipe. In creating these meals, the Healthy Eating Plate (46) and the recommended portion sizes for each ingredient were considered. Another example of a balanced healthy Spanish meal could be the following: Main course: Pink hummus (chickpeas, cooked beets, tahini, lemon juice, olive oil, garlic, salt) with carrot sticks; Second course: Stuffed Roasted tomatoes with brown rice and grilled tuna with olive oil. Dessert: Seasonal fruit. Since Türkiye people usually consume bread along with their meals, the calories in the meals were slightly reduced. An example of a balanced healthy Turkish dinner meal based on the updated Mediterranean diet food pyramid (41), could include: Ezogelin soup (tomato paste, olive oil, flour, water, salt, lemon juice, onions, bulgur, red lentils, dried mint), lemon chicken (chicken breast, olive oil, rosemary, black pepper, salt, butter, garlic, lemon, walnuts, parsley), sautéed beetroot salad (beets, olive oil, walnuts, salt, dill, yoghurt) and einkorn bread.

**Table 4:** A Spanish daily meal plan example.

Dish name	Ingredients (standard portion for an adult)	Nutritional composition	Food components	Recipe
Breakfast: *Yoghurt plain with blueberries and oatmeal*				
Yoghurt plain with blueberries and oatmeal	Yoghurt plain, 125 g Blueberries, 50 g Oatmeal, 30 g	Kcal: 195.50 Protein: 11.53 g Fat: 2.32 g Carbohydrates: 27.14 g	Dairy, Cereals, Fruit	Add the yogurt to a bowl. Then add the blueberries and oat flakes. Mix all ingredients.
Snack: *Turkey, apple and rocket sandwich*				
Turkey, apple and rocket sandwich	Whole meal bread, 60 g Sliced turkey, 40 g Apple, 40 g Arugula, 10 g Olive oil, 10 mL	Kcal: 301.17 Protein: 13.64 g Fat: 11.89 g Carbohydrates: 31.90 g	White meat, Cereals, Fruit, Raw vegetables Cooked vegetables	Slice the bread crosswise, add a drizzle of olive oil, the turkey and the washed rocket to one half of the bread. Wash the apple and cut into thin slices. Add the apple to the sandwich and serve.
Lunch: *Stewed lentils with vegetables and rice. Orange*				
Stewed lentils with vegetables and rice	Lentils, 60 g Onion, 60 g Carrot, 100 g Green bell pepper, 40 g Rice, 30 g Garlic, 1.6 g Tomato sauce, 50 g Extra virgin olive oil, 15 g Salt, 0.8 g Black pepper, 0.8 g	Kcal: 362.12 Protein: 8.39 g Fat: 16.11 g Carbohydrates: 42.64 g	Pulses (Legumes), Lentils, Rice, Soups, Cereals, Cooked vegetables	Wash and peel the vegetables, cut into pieces and fry with fried tomato for two minutes. Add the dried lentils and enough water to cover everything, then cook over medium-low heat for 30 min. Add the rice and cook for another 20 min or until the rice is cooked. Taste and adjust the seasoning to taste.
Orange	Orange, 200 g	Kcal: 65.52 Protein: 1.08 g Fat: 0.72 g Carbohydrates: 11.56 g	Fruit	
Dinner: *Couscous and pomegranate fresh salad. Grilled tuna with sweet potato puree. Apple.*				
Couscous and pomegranate salad	Couscous, 80 g Pomegranate, 70 g Cucumber, 50 g Tomato, 50 g Fresh mint, 10 g Extra virgin olive oil, 10 g	Kcal: 419.20 Protein: 10.75 g Fat: 11.63 g Carbohydrates: 68.18 g	Pasta, Fruit, Raw vegetables	Prepare couscous as directed. Mix with pomegranate seeds, cucumber, tomato, and fresh mint. Dress with olive oil and season to taste.
Grilled tuna with sweet potato puree	Tuna, 150 g Sweet potato, 100 g Potato, 50 g Milk, 50 g; Butter, 15 g; Extra virgin olive oil,10 g	Kcal: 591.35 Protein: 37.86 g Fat: 35.14 g Carbohydrates: 28.92 g	Fish or Seafood, Dairy, Tubers	Grill bonito steaks. Boil sweet potatoes and potatoes, mash with milk and butter. Serve the bonito over the puree, drizzled with olive oil.
Apple	Apple, 180 g	Kcal: 89.14 Protein: 0.41 g Fat: 0.41 g Carbohydrates: 19.21 g	Fruit	

Four meals are presented along with their corresponding dishes, nutritional information, food composition and recipe.

Regional food composition databases were used for determining the nutritional composition of dishes in each country, ensuring accuracy and relevance of the nutritional data according to local food consumption patterns. In Turkish dishes, the National Food Composition Database “TURKOMP” (47) and the Nutrition Information System “BeBiS” database (48) developed by the Ministry of Agriculture and Forestry were used. For the Spanish dishes we used the Ciqual Database (Table of Nutritional Composition of Foods) by the French Agency for Food, Environmental and Occupational Health Safety (ANSES) (49).

#### 2.2.2 Technical implementation of the database

**Figure 2:**
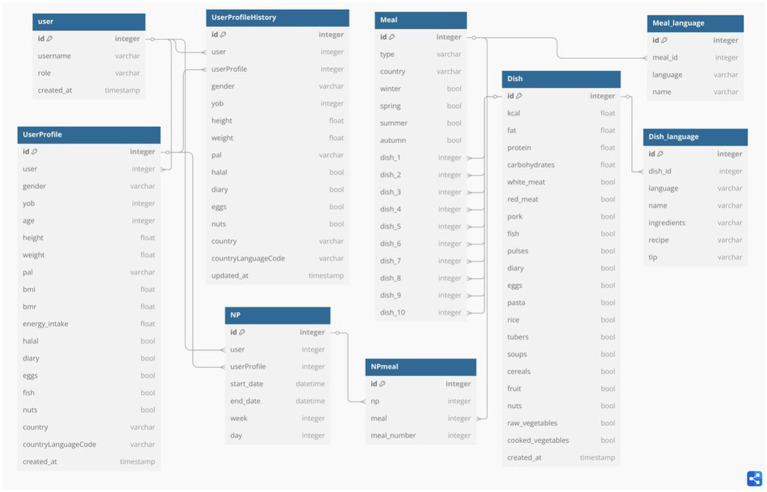
SWITCHtoHEALTHY database schema. Figure 2 presents the SWITCHtoHEALTHY database schema, displaying all tables along with their respective fields, data types, and relationships with other tables. This schema provides a structured overview of how data is organized and interconnected within the system.

The SWITCHtoHEALTHY Database consists of nine tables, with the key ones for the NP generator being the “User Profile,” “Dish,” and “Meal” tables. Additionally, supplementary tables such as “NP” and “NPmeal” facilitate tracking user’s weekly nutritional plans. The “Meal Language” and “Dish Language” tables ensure multilingual accessibility of meal and dish information. Moreover, the “User” table manages authentication by storing user credentials, while the “User Profile History” table records changes made to user profiles. This structured arrangement optimizes data management and facilitates the generation of personalized meal recommendations. Figure 2 illustrates the SWITCHTOHEALTHY database schema.

##### 2.2.2.1 User profile table

All essential user information, including data required by the NP generator, is stored in the “UserProfile” table. This table has a one-to-one relationship with the “User” table, meaning each user has only one profile. As described in section 2.1.3, key mathematical formulas such as BMI, BMR, and DER play a crucial role in our algorithm. To compute these metrics, the system requires fields such as “sex,” year of birth (“yob”), “height,” “weight,” and physical activity level (“PAL”). The values for “Age,” “BMI,” “BMR,” and “energy intake” are automatically calculated and stored in the database. Additionally, Boolean fields like “halal,” “dairy,” “eggs,” “fish,” and “nuts” help track the user’s allergies and preferences. Lastly, with “country” and “countryLanguageCode,” users can specify their preferred country (e.g., Spain, Türkiye) for meal recommendations and select their preferred language for viewing meal details.

##### 2.2.2.2 Meal/dish tables

Meal and Dish tables for the core of the database and have been meticulously designed and underwent numerous modifications and improvements. In this section, we will present the architecture of the Meal and Dish tables. Meals may consist of one or more dishes, granting the freedom to create unique meals (comprising of a single dish) or more complex ones (comprising up to 10 dishes).

In the “Meal” table, we store the dish IDs, which serve as foreign keys to the dishes comprising this meal. We also store information about the seasons. Each meal has four options (Winter, Spring, Summer, Autumn), and for each one, we mark ‘yes’ or ‘no’ to show if the meal is suitable for that season. The “Country” field is crucial for selecting which local cuisine will be presented to the users. Lastly, the “Type” field includes the five meal types associated with the meals: “breakfast,” “morning snack,” “lunch,” “afternoon snack,” and “dinner.”

In the “Dish” table, all the necessary information about calories, fat, protein, and carbohydrates is stored. Additionally, we keep track of food categories included in a specific dish. These categories include “white meat,” “red meat,” “pork,” “fish,” “pulses,” “dairy,” “eggs,” “pasta,” “rice,” “tubers,” “soups,” “cereals,” “fruit,” “nuts,” “raw vegetables,” and “cooked vegetables.” Part of this information is highly important for the NP generator and for the expert-validated rules provided by the nutritionists.

#### 2.2.3 The database

Before running the evaluation of the AINR, we believed it was very crucial to evaluate the database. This way, we could understand the advantages and disadvantages of the database to better analyze the experimental results. The database evaluation was divided into two parts: one for the Spanish meals/dishes database and another one for the Turkish. The procedure of the evaluation was the same for both countries. The primary aspects of the evaluation initially involved checking for the availability of appropriate meals for each season, alongside with allergies and dietary filters. Following this, we examined the caloric, fat and protein ranges of the meals, all of which are necessary for the AINR.

##### 2.2.3.1 Spanish cuisine

The Spanish database consists of a total number of 90 meals. Of these, 20 are breakfasts, 10 are morning snacks, 30 are lunches, 10 are afternoon snacks and 20 are dinners.

As mentioned earlier, the AINR combines these meals to create meal plans. However, in Steps 1 and 2 of the AINR, meals are first filtered based on seasonality and the user profile attributes (allergies, preferences). Thus, to evaluate the database, it is important to know the number of meals in the database per type for each season/attribute because these are the meals that will be used by the AINR in Step 3 for each user, respectively, (based on the season and their profile). Table 5 illustrates the number of Spanish meals per type for each season and user profile attribute (allergy, preferences). We observe that, in the case of milk and nuts allergy, the number of meals is relatively small, and for specific meal types (e.g., breakfast) the count goes down to zero. This fact explains some of the issues that are discussed in the experimental results (section 3.3).

**Table 5:** Available appropriate Spanish meals for each season according to allergies and preferences.

Allergies/Preferences	Breakfasts	Morning Snacks	Lunches	Afternoon Snacks	Dinners
	Available appropriate meals for Winter				
No	14	7	21	6	15
Milk	0	3	11	2	9
Eggs	12	7	16	6	8
Fish	14	7	15	6	9
Nuts	11	4	8	2	13
Halal	13	7	20	6	13
	Available appropriate meals for Autumn				
No	16	8	21	6	17
Milk	0	4	9	2	11
Eggs	13	8	18	6	10
Fish	16	8	15	6	10
Nuts	13	4	19	2	15
Halal	15	8	21	6	15
	Available appropriate meals for Summer				
No	18	7	23	10	19
Milk	0	4	11	3	13
Eggs	15	7	18	10	11
Fish	18	7	12	10	10
Nuts	15	3	21	5	17
Halal	17	7	23	10	17
	Available appropriate meals for Spring				
No	15	9	25	10	19
Milk	0	5	12	3	13
Eggs	13	9	17	10	11
Fish	15	9	14	10	10
Nuts	12	4	22	5	17
Halal	14	9	24	10	17

Additionally, *calories*, *fat* and *protein* levels are key parameters utilized in the AINR scoring mechanism. Understanding the ranges of these three attributes in the existing meals in the database can significantly help evaluate the experimental results. Table 6 illustrates the mean, standard deviation (std) and confidence intervals (CI) of caloric, fat and protein ranges of the Spanish meals for each season. Fat and protein values are expressed as percentages of total caloric intake. In terms of calories, we observe that 70% of the NPs fall within the range of 2,000-2,600, while 99% of them are within the range of 1,600 to 3,000, for all seasons. This indicates that the database is robust and covers a wide spectrum of calorie levels. Moreover, fat and protein values appear to align with the expert-validated rules presented in Table 2.

**Table 6:** Caloric, fat and protein ranges for all possible Spanish NPs (Nutritional Plans) for each season.

	Mean	Std	68% CI	95% CI	99% CI
	Winter: 185,220 NPs				
Calories (Kcal)	2,340	258	2,083-2,597	1,833-2,847	1,674-3,006
Fat %	38	5	33–43	29–47	26–50
Protein %	18	4	15–22	11–26	9–28
	Spring: 641,250 NPs				
Calories (Kcal)	2,318	263	2,056-2,579	1,803-2,832	1,641-2,995
Fat %	37	5	33–42	28–46	26–49
Protein %	18	4	15–22	11–26	8–28
	Summer: 550,620 NPs				
Calories (Kcal)	2,326	263	2,065-2,588	1,811-2,842	1,649-3,004
Fat %	37	5	33–42	29–46	26–49
Protein %	18	4	15–22	11–26	8–28
	Autumn: 274,176 NPs				
Calories (Kcal)	2,367	266	2,102 – 2,631	1,845-2,889	1,681-3,053
Fat %	37	4	33–42	28–46	26–49
Protein %	18	4	15–22	11–26	9–28

##### 2.2.3.2 Turkish cuisine

The Turkish database consists of a total number of 90 meals. Of these 20 are breakfasts, 10 are morning snacks, 28 are lunches, 12 are afternoon snacks and 20 are dinners.

Correspondingly, to Table 5 for the Spanish meals, Table 7 illustrates the number of Turkish meals per type for each season and user profile attribute (allergy, preferences). Overall, there is a plethora of meal options for almost every season and every user profile attribute (allergies, preferences). The number of meals is slightly reduced for the milk allergy.

**Table 7:** Available appropriate Turkish meals for each season according to allergies and preferences.

Allergies/Preferences	Breakfasts	Morning Snacks	Lunches	Afternoon Snacks	Dinners
	Available appropriate meals for Winter				
No	16	6	14	9	13
Milk	4	1	8	6	5
Eggs	6	6	8	9	10
Fish	16	6	14	9	11
Nuts	16	4	14	4	13
Halal	16	6	14	9	13
	Available appropriate meals for Autumn				
No	16	6	13	10	12
Milk	4	1	7	7	4
Eggs	6	6	10	10	10
Fish	16	6	13	10	10
Nuts	16	4	12	4	12
Halal	16	6	13	10	12
	Available appropriate meals for Summer				
No	16	8	22	8	14
Milk	5	1	8	4	6
Eggs	5	8	16	8	10
Fish	16	8	22	8	12
Nuts	15	7	21	5	13
Halal	16	8	22	8	14
	Available appropriate meals for Spring				
No	15	5	16	6	14
Milk	6	0	4	2	5
Eggs	5	5	11	6	10
Fish	15	5	16	6	12
Nuts	15	4	16	4	13
Halal	15	5	16	6	14

Correspondingly, to Table 6 for the Spanish meals, Table 8 illustrates the mean, standard deviation (std) and confidence intervals (CI) of caloric, fat and protein ranges of the Turkish meals for each season. Fat and protein values are expressed as percentages of total caloric intake. In the Turkish database, the caloric values are observed to be relatively low. Across all seasons, 70% of NPs fall within the range of 1,500 to 2,000 calories, while 99% of them range from 1,100 to 2,400. This could pose challenges in generating meals from users requiring more than 2,400 calories. However, the fat and protein values seem to follow the expert-validated rules presented in Table 2.

**Table 8:** Caloric, fat and protein ranges for all possible Turkish NPs (Nutritional Plans) for each season.

	Mean	Std	68% CI	95% CI	99% CI
	Winter: 157,248 NPs				
Calories (Kcal)	1,821	233	1,589-2,053	1,364-2,278	1,220-2,421
Fat %	40	8	33–49	25–56	20–61
Protein %	16	3	13–19	10–22	8–24
	Spring: 100,800 NPs				
Calories (Kcal)	1,685	225	1,461-1,910	1,244-2,126	1,106-2,265
Fat %	40	8	33–48	25–56	20–61
Protein %	16	3	13–19	10–21	9–23
	Summer: 315,392 NPs				
Calories (Kcal)	1,742	252	1,491-1,993	1,247-1,237	1,092-2,392
Fat %	38	9	29–47	20–56	15–61
Protein %	15	3	12–17	9–20	8–22
	Autumn: 149,760 NPs				
Calories (Kcal)	1,808	133	1,577-2,039	1,352-2,264	1,109-2,407
Fat %	40	8	32–48	25–56	20–60
Protein %	16	3	12–19	9–22	7–24

## 3 Results

Before conducting the evaluation of the recommendation system, we considered several main factors, which are also useful when the system runs in real-time. These factors can be categorized as follows: (a) validation of filtering accuracy regarding country, allergies, preferences, and seasonality (b) evaluation of diversity and food group variety (c) evaluation of accuracy in terms of suggested calories, macronutrients (fat, protein), and fruits and vegetables. To evaluate the system across these three main categories and their subcategories, several user profiles were generated. The system evaluation will also offer valuable insight into the expected performance of the system in real-world conditions.

### 3.1 Generated users profiles

In our real-time system, we offer two country options (Spain and Türkiye) alongside with two sex choices (male and female). To determine the total number of generated user profiles, we deemed it prudent to create 1,000 profiles for each country and sex, resulting in a total of 4,000 profiles. According to meticulously researched data on major food allergies in Europe, presented in (50), we incorporated allergies into our sample based on the following percentages: dairy (6%), eggs (2.5%), fish (2%), and nuts (2.5%). Regarding preferences, our system supports the “Halal” choice, meaning that we should not include “pork” in our meal proposals; we estimated that 3% of users in Spain and 50% of users in Türkiye – where Islam, a religion that prescribes a halal diet, is predominant – would choose this option. It is important to note that we prevent all combinations of different allergies and the “Halal” preference in our sample. Thus, user profiles may have only one allergy or preference.

To populate the generated user profiles, another crucial aspect was to predefine their physical characteristics. The user’s physical characteristics (sex, age, height, weight) inputs lead to their Body Mass Index (BMI) and Basal Metabolic Rate (BMR). The BMI and BMR, combined with the PAL (Physical Activity Level), determines the Daily Energy Requirements (DER) of a user (see Section 2.1.3). DER is the only input our AINR requires from the users to sort the NPs. Instead of manually defining each user’s physical characteristics, we opted to directly generate their DER. To ensure the realism of the results, we conducted research to find appropriate recommended energy requirement levels (51, 52). The mathematical formulas used to calculate the DER of the generated user profiles are detailed above.


DER=1.000×f(x)

$$\text{where}\;f(x)=\frac{1}{\sigma \times \sqrt{2\pi }}\times e^{-\frac{1}{2}\times {\left(\frac{x-\mu }{\sigma }\right)}^{2}},$$

$$\mu =2,5\;\text{and}\;\sigma =\frac{1}{3},\text{for}\;\mathrm{men},\;\text{and}$$

$$\mu =2\;\text{and}\;\sigma =\frac{4}{15},\text{for women}$$

**Figure 3:**
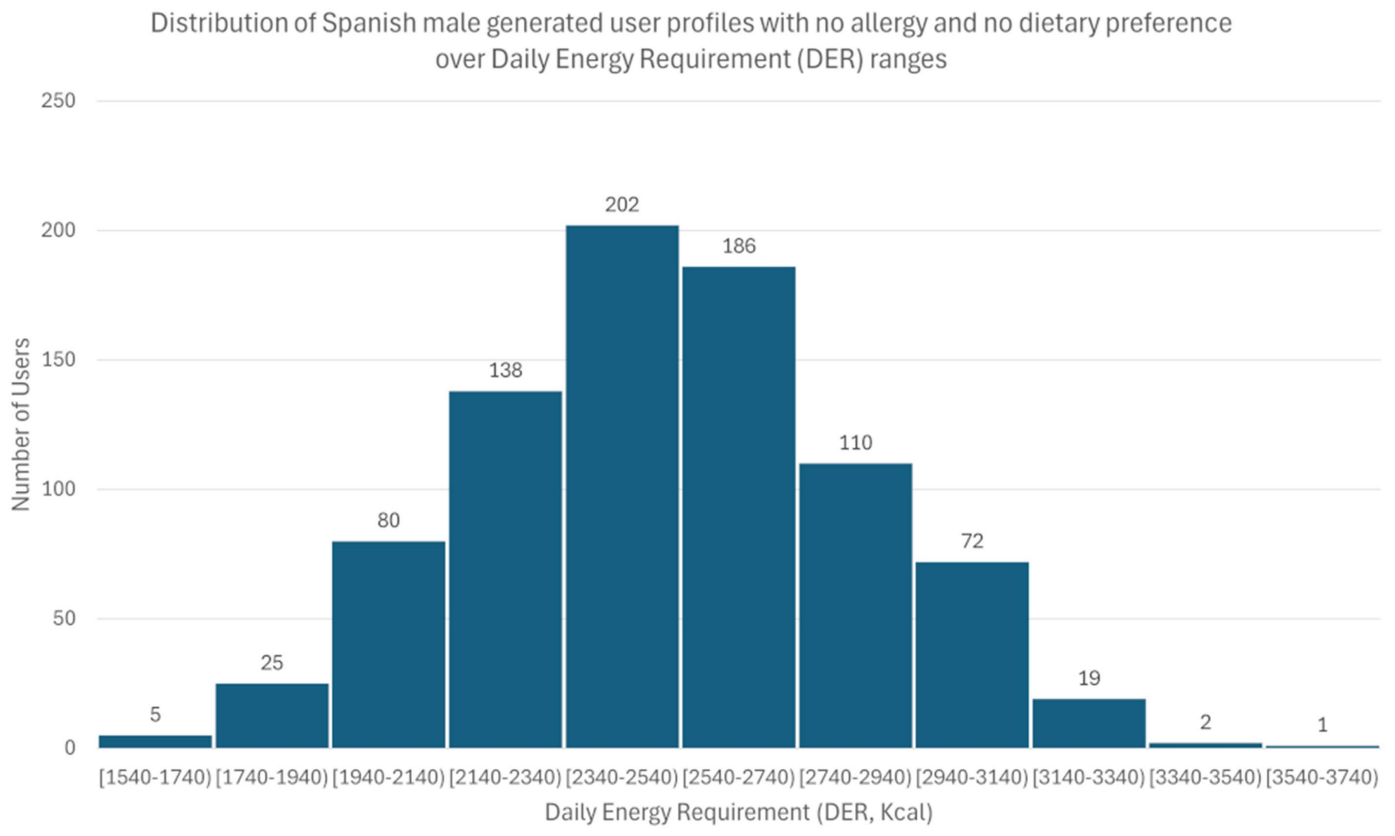
Distribution of Spanish male generated user profiles with no allergy and no preferences over Daily Energy Requirement (DER) ranges. The *x*-axis represents DER ranges in kcal, while the *y*-axis indicates the number of generated user profiles with no allergy and no preferences.

**Figure 4:**
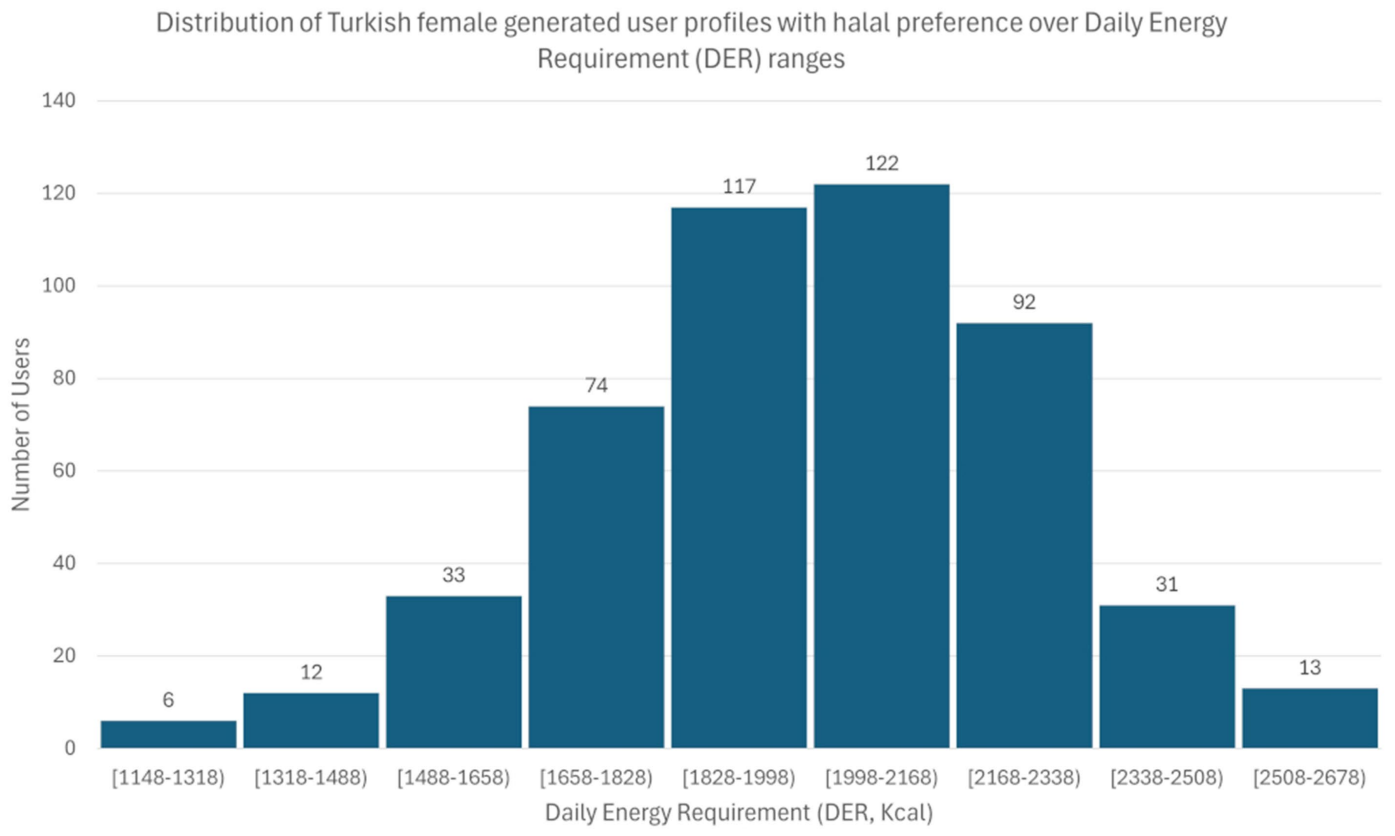
Distribution of Turkish female generated user profiles with halal preference over Daily Energy Requirement (DER) ranges. The *x*-axis represents DER ranges in kcal, while the *y*-axis indicates the number of generated user profiles with halal preference.

The distribution was applied separately to each one of the user profiles categories. An example of the distribution of Spanish male generated user profiles with no allergy and no preferences can be seen in Figure 3. In Figure 4, we can see the distribution of Turkish female generated user profiles with halal preference. In both examples, we observe that the generated users follow the normal distribution formulas presented above.

### 3.2 Evaluation method

The experiment was conducted for all 4,000 generated user profiles, with the goal of generating 7 daily NP proposals for each user, totaling 28,000 daily NPs. Each daily NP consists of five meals: breakfast, morning snack, lunch, afternoon snack, and dinner. As described, the AINR only requires the user’s DER, country and allergy/preferences to generate recommendations. Initially, we verified that filtering by country, allergies/preferences, and seasonality was 100% accurate. Then, we evaluated how many generated user profiles met the diversity and food group intake rules necessary to receive a weekly meal plan proposal (exactly 7 daily NPs). Only profiles that met these criteria were included in the results. Finally, we evaluated the AINR’s performance based on its accuracy in suggesting total calories, macronutrients (fat, protein), and fruit and vegetable intake.

### 3.3 Experimental results and discussion

As a first step, we tested our algorithm to filter meals with maximum precision. Users should receive meal proposals from their preferred country, avoiding meals that conflict with their allergies and preferences, and receive meal proposals that follow seasonality. To reiterate, the country filter selects meals for either Spain or Türkiye cuisine, the allergies filter excludes meals containing dairy, eggs, fish, or nuts ingredients, the preferences filter excludes meals containing pork as an ingredient, and the seasonality filter selects meals compatible with the season (Winter, Spring, Summer, Autumn). We conducted a thorough examination of our algorithm across all categories and achieved 100% accuracy in each one of them.

As a next step, we tested the number of generated user profiles who managed to receive exactly 7 daily NPs, according to our diversity and food group intake criteria described in section 2.1.4. Specifically, Tables 9, 10 illustrate the percentages of profiles who managed to receive a complete weekly meal plan consisting of 7 daily NPs, with results separated for Spanish and Turkish profiles, male and female, showing how each generated user profile category succeeded in all seasons. This approach ensures that the evaluation captures how well the dietary plans adhere to nutritional rules throughout the year, considering possible seasonal changes in food availability.

**Table 9:** Acceptable weekly plans accuracy for male and female Spanish generated Users for each season.

Allergies/Preferences	Users/Weekly plans	Acceptable weekly plans (%)							
		Winter		Spring		Summer		Autumn	
		Male	Female	Male	Female	Male	Female	Male	Female
None	840	98.57%	96.90%	100.00%	100.00%	100.00%	99.88%	97.74%	98.45%
Dairy	60	0.00%	0.00%	0.00%	0.00%	0.00%	0.00%	0.00%	0.00%
Eggs	25	96.00%	100.00%	100.00%	100.00%	100.00%	100.00%	100.00%	100.00%
Fish	20	100.00%	100.00%	100.00%	100.00%	100.00%	100.00%	100.00%	100.00%
Nuts	25	0.00%	0.00%	0.00%	0.00%	0.00%	0.00%	0.00%	0.00%
Halal	30	96.67%	96.67%	100.00%	100.00%	100.00%	100.00%	93.33%	100.00%
Total	1,000	**90.10%**	**88.80%**	**91.50%**	**91.50%**	**91.50%**	**91.40%**	**89.40%**	**90.20%**

**Table 10:** Acceptable weekly plans accuracy for male and female Turkish generated Users for each season.

Allergies/Preferences	Users /Weekly plans	Acceptable weekly plans (%)							
		Winter		Spring		Summer		Autumn	
		Male	Female	Male	Female	Male	Female	Male	Female
None	840	100.00%	100.00%	100.00%	100.00%	100.00%	100.00%	93.78%	96.22%
Dairy	60	0.00%	0.00%	0.00%	0.00%	0.00%	0.00%	0.00%	0.00%
Eggs	25	48.00%	80.00%	100.00%	100.00%	100.00%	100.00%	56.00%	44.00%
Fish	20	100.00%	100.00%	100.00%	100.00%	100.00%	100.00%	35.00%	40.00%
Nuts	25	100.00%	100.00%	100.00%	100.00%	100.00%	100.00%	100.00%	100.00%
Halal	30	100.00%	100.00%	100.00%	100.00%	100.00%	100.00%	92.40%	98.00%
Total	1,000	**92.70%**	**93.50%**	**94.00%**	**94.00%**	**94.00%**	**94.00%**	**85.50%**	**89.00%**

As a final step of the evaluation method, caloric accuracy as well as the fat, protein, and fruits and vegetables accuracy are illustrated in Table 11. This table shows results for male and female users for each of the two countries (Spain, Türkiye). It is essential to note here that each user is evaluated based on four weekly meal plans, one for each season (Winter, Spring, Summer, Autumn). For example, for the 60 Spanish male users with a milk protein allergy the total number of corresponding weekly plans evaluated is not 60 but 240 (each of the 60 users has 4 different weekly plans – one per season). “Acceptable weekly plans” corresponds to the number of weekly plans consisting of exactly 7 daily NPs that also follow all the nutritional rules, and “% total weekly plans” provides the percentage of these plans to the total weekly plans (also including not acceptable ones). The algorithm manages to achieve 98% average accuracy in calories and over 90% average accuracy in macronutrients (protein, fat) and fruits and vegetables for Spanish male and female users and for Turkish female users. Lower percentages were recorded for Turkish male users, both in terms of caloric and macronutrient accuracy, aligning with the findings from the Turkish database analysis discussed in Section 2.2.3.

**Table 11:** Caloric, macronutrients (protein, fat) and fruits and vegetables accuracy for (A) Spanish male (B) and Spanish female (C) Turkish male and (D) and Turkish female generated user profiles.

User Allergies/Preferences	Users	Corresponding weekly plans	Acceptable weekly plans	% Total weekly plans	Corresponding daily plans	Mean caloric agreement	Fat within range	Protein within range	Fruit & Veg within the range
(A) Recommender performance for Male generated users using the Spanish meals/dishes Database									
None	840	3,360	3,329	99%	23,303	98%	94%	97%	100%
Milk Protein allergy	60	240	0	0%	0	–	–	–	–
Egg allergy	25	100	98	98%	686	93%	99%	92%	100%
Fish/Seafood allergy	20	80	80	100%	560	97%	96%	96%	100%
Nut allergy	25	100	0	0%	0	–	–	–	–
Halal preference	30	120	117	98%	819	99%	91%	96%	100%
Total/Weighted avg.:	**1,000**	**4,000**	**3,624**	**91%**	**25,368**	**98%**	**94%**	**97%**	**100%**
(B) Recommender performance for Female generated users using the Spanish meals/dishes Database									
None	840	3,360	3,321	99%	23,247	98%	91%	94%	100%
Milk Protein allergy	60	240	0	0%	0	-	-	-	-
Egg allergy	25	100	99	99%	693	95%	82%	85%	100%
Fish/Seafood allergy	20	80	80	100%	560	97%	89%	90%	100%
Nut allergy	25	100	0	0%	0	-	-	-	-
Halal preference	30	120	119	99%	833	96%	88%	91%	100%
Total/Weighted avg.:	**1,000**	**4,000**	**3,619**	**90%**	**25,333**	**98%**	**91%**	**93%**	**100%**
(C) Recommender performance for Male generated users using the Turkish meals/dishes Database									
None	370	1,480	1,457	98%	10,199	88%	88%	69%	100%
Milk Protein allergy	60	240	0	0%	0	-	-	-	-
Egg allergy	25	100	76	76%	532	76%	71%	20%	100%
Fish/Seafood allergy	20	80	67	84%	469	89%	86%	53%	100%
Nut allergy	25	100	100	100%	700	86%	73%	61%	100%
Halal preference	500	2,000	1,962	98%	13,734	87%	88%	67%	100%
Total/Weighted avg.:	**1,000**	**4,000**	**3,662**	**92%**	**25,634**	**87%**	**87%**	**66%**	**100%**
(D) Recommender performance for Female generated users using the Turkish meals/dishes Database									
None	370	1,480	1,466	99%	10,262	98%	97%	92%	100%
Milk Protein allergy	60	240	0	0%	0	-	-	-	-
Egg allergy	25	100	80	80%	560	93%	87%	74%	99%
Fish/Seafood allergy	20	80	68	85%	476	98%	98%	84%	100%
Nut allergy	25	100	100	100%	700	96%	94%	84%	100%
Halal preference	500	2,000	1,990	100%	13,930	98%	96%	93%	100%
Total/Weighted avg.:	**1,000**	**4,000**	**3,704**	**93%**	**25,928**	**98%**	**96%**	**92%**	**100%**

Our results demonstrate that the AINR successfully provided weekly balanced personalized meal plans for most of the generated user profiles (approximately 90% on average), adhering to diversity and food group variety guidelines. For these profiles, the AINR achieved 98% accuracy in energy intake and over 90% average accuracy in macronutrient (fat, protein) and fruit and vegetable distribution. However, challenges arose for certain user profiles that was not able to receive weekly meal proposals, primarily due to limitations in the database related to two specific allergies: milk and nuts, as described in Section 2.2.3. Notably, for nut allergies, the AINR was able to generate weekly meal plans for Turkish users, unlike Spanish users, because the Turkish database was provided with a wider range of nut options.

Augmenting the database with additional meals and dishes could help overcome the challenges faced in both cases by addressing the issues mentioned earlier. The influx of new options will enable the AINR to better cater to user allergies, while also enhancing the accuracy of caloric and macronutrient suggestions, particularly for Turkish male users. Furthermore, meals that can act as substitutes for dairy-based meals could be introduced along with extra filtering in the food intake rules to safeguard users against excessive consumption of processed foods. Our future research plans include extending the AINR to be able to produce meal planning for whole families, instead of individual users, considering common meal suggestions wherever possible. Finally, a key research goal is to test the proposed system with real users in the context of SWITCHtoHEALTHY intervention (53).

## 4 Conclusion

In conclusion, our AI-based nutrition recommendation system represents a promising solution to the modern challenges of maintaining a balanced diet amidst unhealthy dietary intake trends. By leveraging expert-validated dishes and meals from Spanish and Turkish cuisines, the system offers personalized weekly meal plans tailored to users’ nutritional needs and preferences. Through a meticulous five-step process, including filtering, sorting, and ensuring appropriate food group intake and diversity, the system demonstrates effectiveness in providing accurate recommendations. Statistical analysis and algorithmic validation confirm the system’s proficiency across various domains, including filtering accuracy, diversity, food group intake, and accuracy in suggested calories and macronutrients. With experiments conducted on 4,000 generated user profiles, achieving accuracy rates exceeding 90%, our system holds promise in facilitating more balanced dietary habits for individuals in today’s fast-paced world. Despite these promising results, the system has certain limitations, such as constraints related to the underlying food database and the need for validation with real users. However, the main strengths of our work lie in the high accuracy achieved across key nutritional metrics and the system’s ability to provide culturally relevant and allergy-aware meal plans. These aspects underline the significance and practical potential of the proposed approach while identifying clear directions for future research.

## Data Availability

The raw data supporting the conclusions of this article will be made available by the authors, without undue reservation.
